# mHealth for eye care: what is possible?

**Published:** 2022-06-07

**Authors:** Priya Morjaria, Jessica Massie

**Affiliations:** 1 Assistant Professor and Public Health Optometrist: London School of Hygiene & Tropical Medicine and Head of Global Programme Design: Peek Vision, UK.; 2 Freelance Global Eye Health Consultant and Public Health Optometrist, Australia.

**As people's access to cellphones and mobile internet grows, how can mobile health (mHealth) interventions be used to improve patient outcomes in eye health care?**

**Figure:**
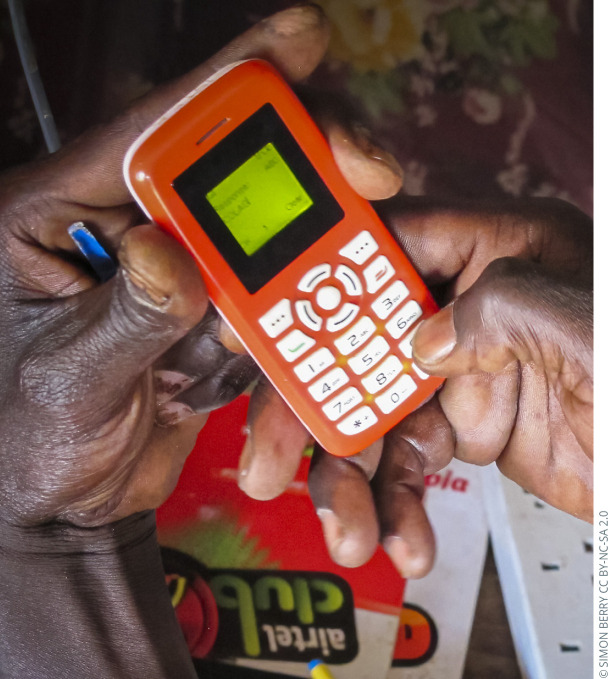
Cellphones can help to empower people to look after their eyes. **ZAMBIA**

Mobile health (mHealth) interventions have become increasingly popular in health care. mHealth refers to the use of any mobile device, but particularly cellphones (also known as mobile phones), to support the achievement of health objectives.

Many mHealth interventions have been implemented in eye care, with the aim to:

- promote attendance at appointments
- promote adherence to medication
- provide eye health education
- increase access to eye care.

## Attendance at appointments

Mobile health (mHealth) interventions can be used to remind patients affected by chronic or long-term eye conditions, such as glaucoma or diabetic retinopathy, to attend their appointments. There are many different ways of sending patients reminders via their cellphones:

- short message service (SMS) or text messages
- voice messages
- automated voice calls (the person answering the phone hears a pre-recorded message)
- interactive automated phone calls (the person answering hears a message and can press numbers on the keypad to interact with the system)
- telephone hotlines (volunteers call patients to remind them of their appointments).

In one example, automated telephone calls were used in the USA to remind patients about their diabetic retinopathy screening appointments; patients were also able to reschedule their appointment if needed. This had positive results, including significant improvement in appointment adherence.^1^ Patients who received the phone call were more likely to schedule their appointment and complete their appointment, compared with usual care.

## Adherence to medication

In patients with glaucoma, adherence to medication is vital for controlling intraocular pressure and slowing down disease progression. However, studies have indicated that adherence is not sufficient. To address this, a smartphone application was developed in Nigeria that patients could use to set up reminders to instil their glaucoma eye drops. Patients had better adherence to medication when using the application. One challenge that patients faced was unreliable electricity supply, which made it difficult to keep their phones charged and working.^2^

## Patient education

The World Report on Vision emphasises the need to improve communication between patients and practitioners in order to facilitate decision-making and counselling. This is especially important in the context of the move towards integrated people-centred eye care.^3^

A web-based service called DiasNet has been implemented in Denmark and the UK. It is used by doctors and patients to improve education and communication in diabetes care.^4^ This tool allows patients to see for themselves the changes in retinal lesions from one appointment to next, because of changes in their lifestyle and glycaemic control. Patients can then experiment with their own data and retrospectively adjust insulin doses, meal sizes, etc., allowing them to learn how to better manage their diabetes.

A study carried out in China used the WeChat communications app to decrease the anxiety experienced by parents of children with congenital cataract. This involved sharing health information with parents by sending links to online videos. As a result, parents’ satisfaction and understanding increased and their levels of anxiety decreased.^5^

## Improving access to eye care

In India and Kenya, Peek software was used to screen and identify children who needed to visit an eye specialist or wear spectacles. SMS and voice messages were used to inform parents about the need for their children to visit an eye specialist or receive spectacles, and why this was important. In Kenya, the adherence to referral was twice as high in the group where parents received an SMS reminder compared to the group where parents did not receive such a reminder. The proportion of pupils identified as having visual impairment who attended their hospital referral was also significantly higher in the group that received the SMS reminders.^6^ In India, where voice messages were sent to parents to provide health education, the compliance with spectacle wear in children was higher than in any previous study: an average of 53%.^7^

## Implementing mHealth solutions

With increasing access to cellphones and smartphones, and with mobile internet connectivity growing in sub-Saharan Africa and Asia,^8^^,^^9^ the potential for mHealth to be implemented within eye health care is growing. However, there is a need for more evidence from low- and middle-income countries on the impact of mHealth interventions in eye care. It is also important to acknowledge that women and people in rural areas still tend to be left behind in terms of cellphone use and mobile internet access.^10^

Eye health practitioners are encouraged to explore the use of mHealth within their setting. However, before implementing a new mHealth intervention, it is important to:

- understand the evidence which supports the use of this intervention
- ensure that the intervention is locally acceptable and/or can be adapted
- make provision for people with disabilities to ensure they can also benefit from the intervention
- check that the intervention complies with local legislation and regulations, including the protection of personal data.
